# Effects of Maternal Chromium Restriction on the Long-Term Programming in MAPK Signaling Pathway of Lipid Metabolism in Mice

**DOI:** 10.3390/nu8080488

**Published:** 2016-08-10

**Authors:** Qian Zhang, Xiaofang Sun, Xinhua Xiao, Jia Zheng, Ming Li, Miao Yu, Fan Ping, Zhixin Wang, Cuijuan Qi, Tong Wang, Xiaojing Wang

**Affiliations:** 1 Key Laboratory of Endocrinology, Ministry of Health, Translational Medical Center, Department of Endocrinology, Peking Union Medical College Hospital, Peking Union Medical College, Chinese Academy of Medical Sciences, Beijing 100730, China; zhangqian6088@pumch.cn (Q.Z.); zhengjia_pumch@sohu.com (J.Z.); liming_pumch@sohu.com (M.L.); yumiao_pumch@sohu.com (M.Y.); fanping_pumch@sohu.com (F.P.); wangzhixinpumch@sohu.com (Z.W.); qicuijuan_pumch@sohu.com (C.Q.); wangtong_pumch@sohu.com (T.W.); wangxiaojingpum@sohu.com (X.W.); 2 Department of Endocrinology, The Affiliated Hospital of Qingdao University, Qingdao 266003, China; sunxiaofangpu@sohu.com

**Keywords:** DNA methylation, chromium restriction diet, lipid metabolism, MAPK pathway, metabolic programming

## Abstract

It is now broadly accepted that the nutritional environment in early life is a key factor in susceptibility to metabolic diseases. In this study, we evaluated the effects of maternal chromium restriction in vivo on the modulation of lipid metabolism and the mechanisms involved in this process. Sixteen pregnant C57BL mice were randomly divided into two dietary treatments: a control (C) diet group and a low chromium (L) diet group. The diet treatment was maintained through gestation and lactation period. After weaning, some of the pups continued with either the control diet or low chromium diet (CC or LL), whereas other pups switched to another diet (CL or LC). At 32 weeks of age, serum lipid metabolism, proinflammatory indexes, oxidative stress and anti-oxidant markers, and DNA methylation status in adipose tissue were measured. The results indicated that the maternal low chromium diet increased body weight, fat pad weight, serum triglyceride (TG), low-density lipoprotein cholesterol (LDL), tumor necrosis factor-α (TNF-α), malondialdehyde (MDA), and oxidized glutathione (GSSG). There was a decrease in serum reduced/oxidized glutathione (GSH/GSSG) ratio at 32 weeks of age in female offspring. From adipose tissue, we identified 1214 individual hypomethylated CpG sites and 411 individual hypermethylated CpG sites in the LC group when compared to the CC group. Pathway analysis of the differential methylation genes revealed a significant increase in hypomethylated genes in the mitogen-activated protein kinase (MAPK) signaling pathway in the LC group. Our study highlights the importance of the MAPK signaling pathway in epigenetic changes involved in the lipid metabolism of the offspring from chromium-restricted dams.

## 1. Introduction

Recently, studies have implied that nutrition during fetal and neonatal life can have profound effects on lifespan, glucose, and lipid metabolism. Human studies conducted in 1944–1945 revealed that undernutrition during early pregnancy was associated with glucose intolerance and increased serum insulin concentrations later in life (50–58 years-old) [1]. It was the first time that scientists addressed the impact of adverse environmental factors in early life on the occurrence of metabolic diseases in adulthood [2]. Later work confirmed and extended this hypothesis by showing significant opposite correlations between birth weight and the risk of adult obesity [3,4]. One important mechanism believed to be involved in this relationship is DNA methylation. DNA methylation changes normally occur within CpG-rich regions (CpG islands). CpG islands are usually located near the promoter regions of genes. Methylation within the promoter region can negatively affect gene expression [5]. DNA methylation is a key regulator of normal metabolic balance and the occurrence of disease [6,7]. DNA methylation changes are particularly sensitive in “early life window period”. DNA methylation changes occurring in utero may be passed on to offspring and may subsequently lead to metabolic diseases [8].

An increasing number of studies show that chromium (Cr (III)) supplementation is beneficial in maintaining healthy lipid metabolism, regulating appetite, reducing fat mass, and increasing lean body mass [9]. The minimum suggested daily chromium intake is 30 μg. However, the average dietary chromium intake for adults is far below this recommendation in many countries [10,11]. In particular, pregnant women and elderly individuals are more prone to the chromium deficiency [12], due to increased metabolic stress and decreased absorption ratio [13,14]. Vincent et al. report that a chromium-insufficient diet leads to an increase in serum cholesterol, which can be ameliorated by chromium supplementation [15]. A recent study shows that chronic maternal chromium deficiency increases body fat and changes the lipid metabolism in rat pups. The mechanism involved is probably augmented by oxidative stress [16].

We hypothesized that exposure to maternal chromium restriction would have a sustained impact on the methylation of genes involved in lipid metabolism, thus lead to dyslipidemia in mice offspring. To identify this epigenetic alteration, we used a genome-wide DNA methylation approach in adipose tissue and tested whether epigenetic changes were associated with differential gene expression.

## 2. Materials and Methods

### 2.1. Animals Protocol

**Figure 1:**
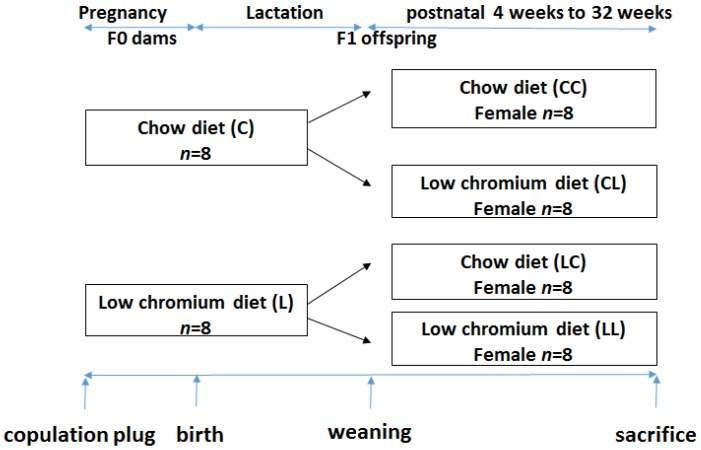
The timeline of the animal experiments.

This study was performed in strict accordance with the recommendations given by the Guide for the Care and Use of Laboratory Animals from the National Institute of Health. The protocol was approved by the Committee on the Ethics of Animal Experiments of Peking Union Medical Hospital (Permit Number: MC-07-6004). All efforts were made to minimize suffering. Seven-week-old C57BL/6J mice (18.5 ± 1.6 g) were acquired from the Institute of Laboratory Animal Science, Chinese Academy of Medical Sciences and Peking Union Medical College (Beijing, China, SCXK-2014-0108). After 1 week of adaptation, virgin female C57BL mice were caged with males (2 females to 1 male) overnight. Copulation was confirmed the next morning by establishing the formation of a vaginal plug. Midnight was considered as day 0 of gestation. Pregnant mothers (*n* = 16) were fed either the control diet (C, *n* = 8) or low chromium diet (L, *n* = 8). The control diet was a casein-based diet formulated on the basis of the American Institute of Nutrition AIN-93G diet and contained 1.19 mg/kg chromium. The low chromium diet (reduced only in chromium) contained 0.14 mg/kg chromium (88.23% of chromium restriction compared to control diet). The concentration of dietary chromium was analyzed using an atomic absorption spectrometer (TAS986, Beijing Persee General Corporation, Beijing, China). All diets were produced by Research Diets (New Brunswick, NJ, USA). On day 1 after birth, the litter sizes of both groups were homogenized to six pups (3 male and 3 female mice), to ensure no nutritional bias between litters. The diets were administered throughout gestation and lactation. All offspring was weaned at 3 weeks of age. Following weaning, the offspring were divided into the following sub-groups: CC (control diet-control diet), CL (control diet-low chromium diet), LC (low chromium diet-control diet), and LL (low chromium diet-low chromium diet, *n* = 8/group, one female pup from each litter was randomly assigned to the experimental groups). The mice were maintained in a light-dark cycle (12 h light and 12 h dark) and were given free access to food and water. Unbalanced maternal nutrition differentially impacted lipid metabolism and phenotypic expression in male and female offspring [17,18]. For this reason, the current study only focused on female offspring. The specific study design is shown in Figure 1. At the end of the experimental period (32 weeks of age), female mice (*n* = 8/group) were sacrificed. After 10 h of fasting, the mice were anesthetized (ketamine 100 mg/kg i.p., Pharmacia and Upjohn Ltd., Crawley, UK), and blood samples were collected from the intraorbital retrobulbar plexus Adipose tissue of the offspring was quickly collected and stored at −80 °C for further analysis.

### 2.2. Serum Chromium Levels

Serum chromium levels in mothers (at weaning) and in the offspring at 32 weeks were determined using an atomic absorption spectrometer (Atomic Absorption Spectrophotometer, Hitachi, Japan).

### 2.3. Measurement of Body Weight and Food Intake

The body weight of the offspring was recorded at birth, 3 weeks, and 32 weeks of age. Food consumption of the offspring was recorded at 32 weeks. Food consumption was quantified by subtracting the amount of food remaining at the end of the week from the total amount of food given at the beginning of the week. The average amount of food consumed per mouse was determined by dividing the total amount consumed by the number of mice.

### 2.4. Measurement of Serum Leptin, Adiponectin and Inflammatory Factors

Serum concentrations of leptin, adiponectin, tumor necrosis factor-α (TNF-α), interleukin-6 (IL-6), monocyte chemotactic protein 1 (MCP-1), and interleukin-1β (IL-1β) were measured using enzyme-linked immunosorbent assay (ELISA, Abcam, Cambridge, MA, USA).

### 2.5. Measurement of Serum Oxidative Stress and Antioxidant Markers

Malondialdehyde (MDA) concentration and reduced/oxidized glutathione (GSH/GSSG) were measured using thiobarbituric acid (TBA) and Thiol Green Indicator fluorometric method (Abcam, Cambridge, MA, USA) as oxidative stress and antioxidant markers, respectively.

### 2.6. Measurement of Serum Total Cholesterol (TC), Triglyceride (TG), High-Density Lipoproterin Cholesterol (HDL), and Low-Density Lipoprotein Cholesterol (LDL)

Serum TC, TG, HDL, and LDL concentrations were determined using an enzyme end-point method via a commercial kit (Roche Diagnostics, GmbH, Mannheim, Germany).

### 2.7. Measurement of Adipose Tissue Weight

At 32 weeks, mice were sacrificed and retroperitoneal, mesenteric, and ovarian fat were carefully removed and weighed. The adiposity index (AI) was computed as followings [19],

(1) $$\text{AI}=\frac{\text{sums of mass from all three fat sources}}{\text{total body mass}}$$

### 2.8. Methyl-DNA Immunoprecipitation and Microarray Hybridization

Fat collected from three CC mice and LC mice was used for methyl-DNA immunoprecipitation (MeDIP) experiment. Genomic DNA (gDNA) was extracted from fat samples using a DNeasy Blood & Tissue Kit (Qiangen, Fremont, CA, USA). The purified gDNA was then quantified and quality was assessed using Nanodrop ND-1000 (NanoDrop Technologies, Wilmington, DE, USA). To perform the MeDIP experiment, first, gDNA was sonicated into random 200–1000 bp pieces. Next, 1 µg of fragmented DNA was used for immunoprecipitation with mouse monoclonal anti-5-methylcytidine (Diagenode, Liege, Belgium) at 4 °C overnight. To recover the immunoprecipitated DNA fragments, anti-mouse IgG magnetic beads (ThermoFisher Scientific, Carlsbad, CA, USA) were added and incubated for an additional 2 h at 4 °C with agitation. Then, immunoprecipitated methylated DNA and input gDNA was labeled with Cy5 and Cy3 fluorophores, respectively. Labelled DNA was hybridized to the Arraystar Mouse ReqSeq Promoter Array (Agilent, Waldbronn, Germany). This array contained all well-characterized RefSeq gene (approximately 22,327 genes) promoter regions (from −1300 bp to 500 bp transcription start sites (TSSs)). Finally, arrays were washed and scanned with an Agilent Scanner G2505C (Agilent Technologies, Waldbronn, Germany). After normalization, methylation peaks in the raw data were analyzed using SignalMap software (Roche Diagnostics, GmbH, Mannheim, Germany). We computed the modified Kolmogorov–Smirnov test on the adjacent probes using sliding windows to predict enriched regions across the array. To separate strong CpG islands from weak CpG islands, promoters were categorized into three levels: high CpG promoters/regions (HCP), intermediate CpG promoters/regions (ICP) and low CpG promoters/regions (LCP) [20].

### 2.9. Differential Methylated Genes Pathway Analysis

To determine the biological meaning to the differentially methylated genes, the subset of methylated genes was analyzed by applying the Gene Ontology (GO) classification system and Kyoto Encyclopedia of Genes and Genomes (KEGG) pathways database using DAVID (Database for Annotation, Visualization and Integrated Discovery) software [21].

### 2.10. Bisulfite Sequencing (BSP)

Bisulfite modification was performed with the EZ DNA Methylation Kit (Zymo Research, Hiss Diagnostics, Germany). The converted DNA was then amplified by PCR with primers detailed in Table 1. Primers were designed using Methyl Primer Express Software version 1.0 (Applied Biosystems, Foster City, CA, USA). PCR products were purified using agarose gels (Invitrogen, Carlsbad, CA, USA) and ligated to the pMD18-T Vector (Takara, Shiga, Japan). The plasmids were then purified using the PureLink Miniprep kit (Invitrogen, Thermo Scientific Inc., Waltham, MA, USA). Positive clones were confirmed by PCR, and a minimum of 10 clones from each mouse (*n* = 8 mice/group) were sequenced using ABI PRISM 7700 Sequence Detection (Applied Biosystems, Foster City, CA, USA). Sequence analysis was performed using QUMA [22].

### 2.11. Quantitative Real Time RT-PCR

The data (*n* = 8/group) were further analyzed for the expression of BSP-validated genes and downstream genes. The total RNA was prepared from fat stored at −80 °C using the Qiagen RNeasy Mini Kit (Qiagen, Germantown, MD, USA). cDNA was synthesized from the reverse transcription of the total RNA using an oligodesoxythymidine primer and the TakaRa RT kit (Takara, Shiga, Japan). The experimental real-time PCR signals were normalized to that of *Gadph* gene. Real-time amplification was performed using the ABI 7900 thermocycler (Applied Biosystems, Foster City, CA, USA). The fold change was calculated using the comparative Ct method. The primer sequences for quantitative real-time PCR are shown in Table 2.

### 2.12. Statistical Analysis

Results are shown as means ± SD; *n* represents the number of mice analyzed. Unpaired Student’s *t* test was used to compare the two groups, and one-way *ANOVA* followed by Tukey’s post hoc test was used when more than two groups were analyzed. For GO and KEGG pathway analysis, Fisher’s exact test was used. A *p* value < 0.05 was considered significant. Prism version 5.0 (GraphPad Software Inc., San Diego, CA, USA) was used for statistical analysis. 

## 3. Results

### 3.1. Maternal Body Weight and Serum Chromium Differences

By the end of lactation, there was no significant difference in maternal body weight between the C dams and L dams (Table 3). As expected, the serum chromium levels were significantly decreased in the L group when compared to the C group (*p* < 0.01, Table 3).

### 3.2. Effects in Offspring

#### 3.2.1. Effects of Maternal Low Chromium Diet on Serum Chromium Level, Body Weight, Food Intake, and Fat Pad Weight in Offspring

**Figure 2:**
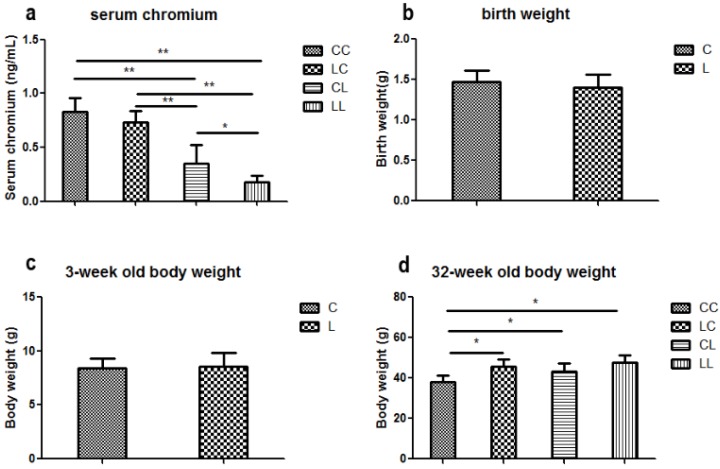

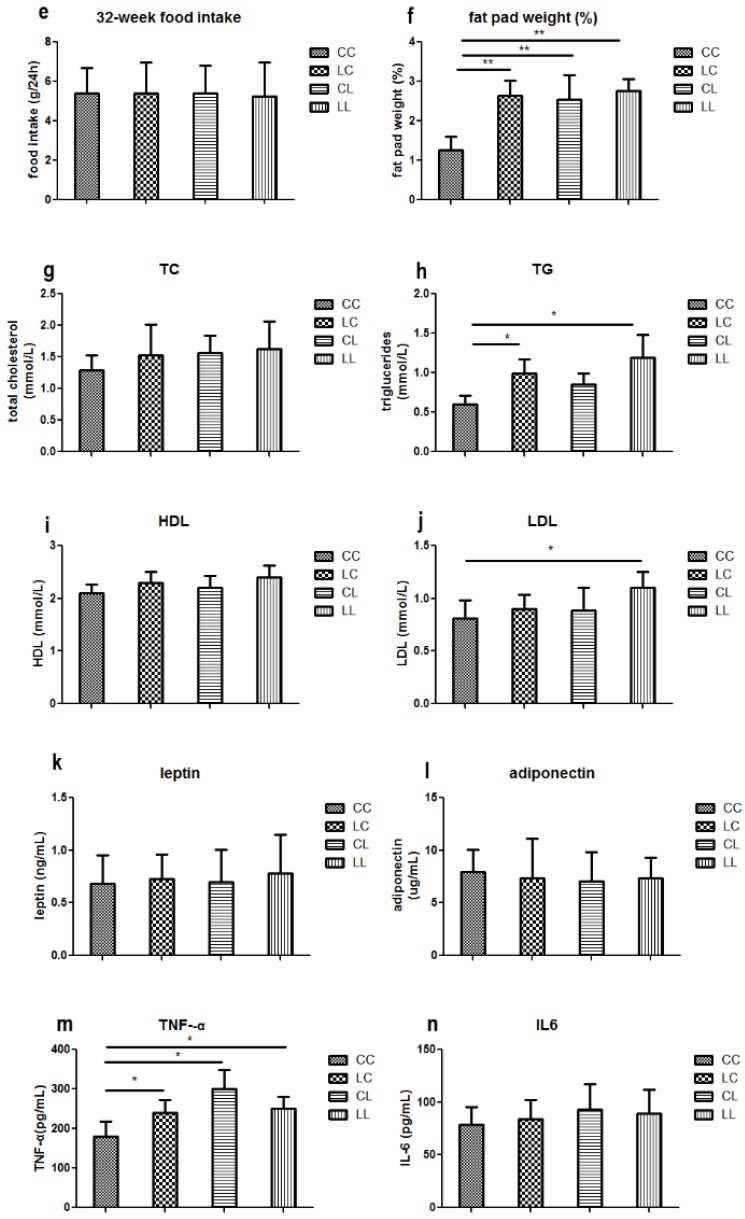

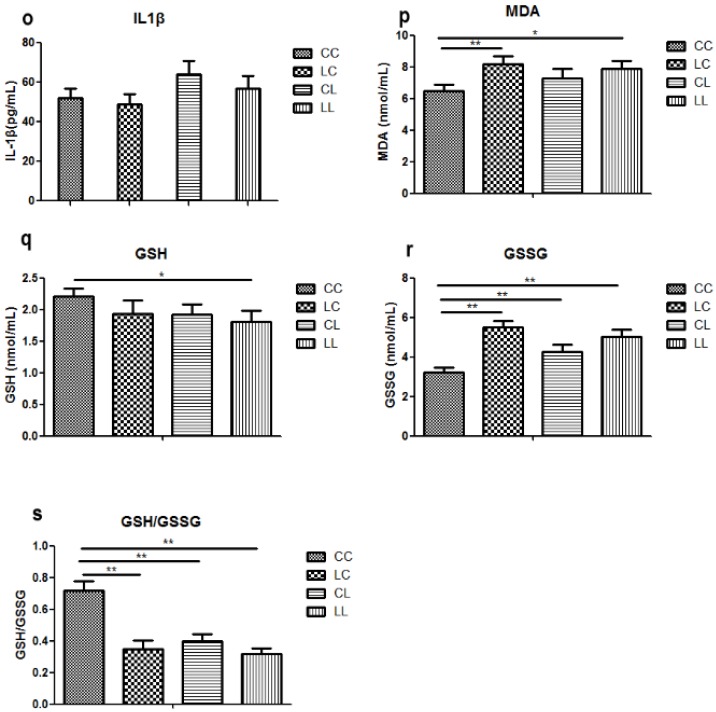
Effects of maternal low chromium diet on serum chromium (**a**); body weight (**d**); food intake (**e**); fat pad weight (**f**); serum total cholesterol (**g**); triglyceride (**h**); HDL (**i**) and LDL (**j**); leptin (**k**); adiponectin (**l**); TNF-α(m); IL-6 (**n**); IL-1β (**o**); MDA (**p**); GSH (**q**); GSSG (**r**); GSH/GSSG (**s**) at 32 weeks of age; birth weight (**b**) and weaning weight (**c**). Values are mean ± SD, *n* = 8 per group. * *p* < 0.05, ** *p* < 0.01.

**Figure 3:**
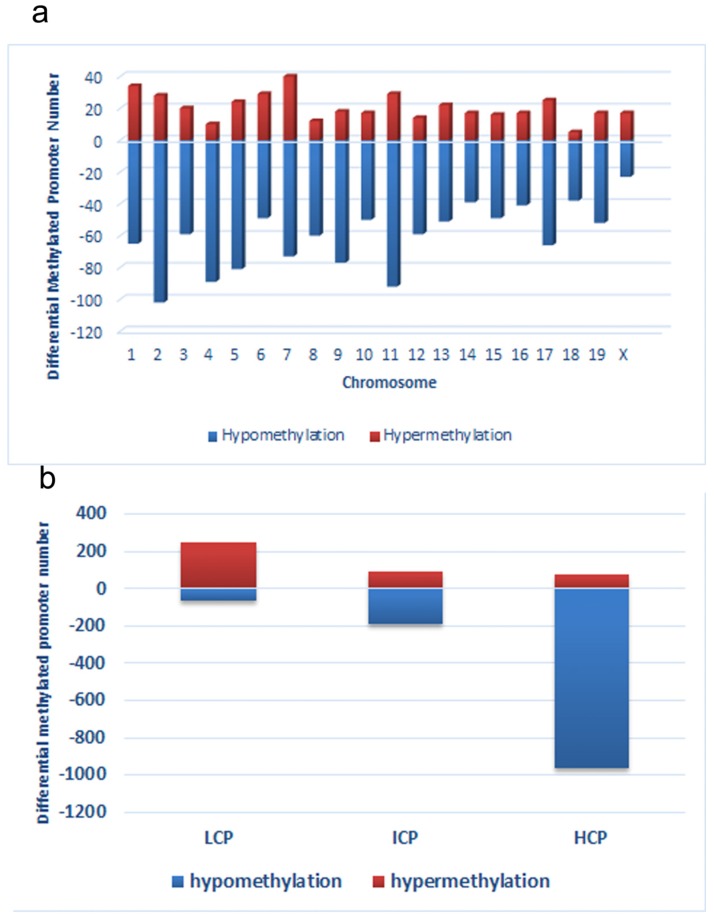
Distribution of differentially methylated promoters on chromosomes (**a**) and promoters with respect to the cytosine polyguanine (CpG) content (**b**), including high CpG promoters/regions (HCP), intermediate CpG promoters/regions (ICP), and low CpG promoters/regions (LCP)in low chromium diet-control diet (LC) group.

At week 32, serum chromium levels were lower in the LL and CL groups when compared to the CC group (*p* < 0.01, Figure 2a). Serum chromium levels in the LC group returned to normal (Figure 2a). The birth weight of L group and C group offspring were comparable (Figure 2b). At weaning, there was still no significant difference in body weight between L and C group (Figure 2c). Although food intake was comparable between the groups (Figure 2e), body weight in the LL group and the CL group were higher than those in CC group at 32 weeks of age (*p* < 0.05, Figure 2d). The body weights of the LC group were higher than those of CC group (*p* < 0.05, Figure 3d). At 32 weeks of age, body fat pad weights were significantly higher in the LL and CL group offspring when compared to those of the offspring from the CC group (*p* < 0.01, Figure 2f). Rehabilitation of low chromium diet dams (LC group) did not correct fat pad weight (*p* < 0.01, Figure 2f).

#### 3.2.2. Effects of Maternal Low Chromium Diet on Serum Lipid Profile in Offspring

Serum TG levels were significantly higher in the LL and LC groups’ offspring when compared to those of the CC group (Figure 2h, *p* < 0.05). LDL in the LL group was higher than the CC group (*p* < 0.05, Figure 2j). However, LDL levels were corrected in the LC group. At 32 weeks of age, serum TC and HDL were not significantly different among the offspring across all groups (Figure 2g,i).

#### 3.2.3. Effects of Maternal Low Chromium Diet on Serum Adiponectin, Leptin, and Pre-Inflammation Cytokines in Offspring

There was no significant differences among the groups in serum adiponectin, leptin, IL-6, and IL-1β values at 32 weeks of age (Figure 2k,l,n,o). Only serum TNF-α levels were significantly higher in the LL, LC, and CL groups when compared to that of the CC group (Figure 2m, *p* < 0.05).

#### 3.2.4. Effects of Maternal Low Chromium Diet on Serum Oxidative Stress and Antioxidant Markers in Offspring

Serum MDA levels were significantly higher in the LC and LL group when compared to that of the CC group at 32 weeks of age (Figure 2p, *p* < 0.01 and *p* < 0.05, respectively). There was no significant difference among the groups in serum GSH (Figure 2q). Serum GSSG levels were significantly higher in LC, CL, and LL groups than that in CC group (Figure 2r, *p* < 0.01). Thus, serum GSH/GSSG ratio was significant lower in LC, CL, and LL groups than CC group (Figure 2s, *p* < 0.01).

#### 3.2.5. Effects of Maternal Low Chromium Diet on Genome-Wide DNA Methylation in Adipose Tissue Collected from Offspring

First, we obtained a CpG promoter methylation profile of the LC group and compared it to that of the CC group (GEO database: GSE82030). This comparison showed that 4.8% (1214 *loci*) of promoters in 1145 genes were hypomethylated in the LC group, particularly on chromosomes 2, 4, 5, 7, 9, 11, and 17, which have more promoters with differential methylation (Peak Score > 2). In contrast, fewer promoters showed a trend toward hypermethylation in the LC group, just 1.8% (411 *loci*) of promoters (Figure 3a). Among the hypomethylated promoters, 963 (79.3%) were located in HCP, 188 (15.5%) in ICP, and 63 (5.2%) in LCP. And among hypermethylated promoters, 73 (17.8%) were located in HCP, 90 (21.9%) in ICP, and 248 (60.3%) in LCP (Figure 3b).

#### 3.2.6. Functional Analysis Using DAVID

**Figure 4:**
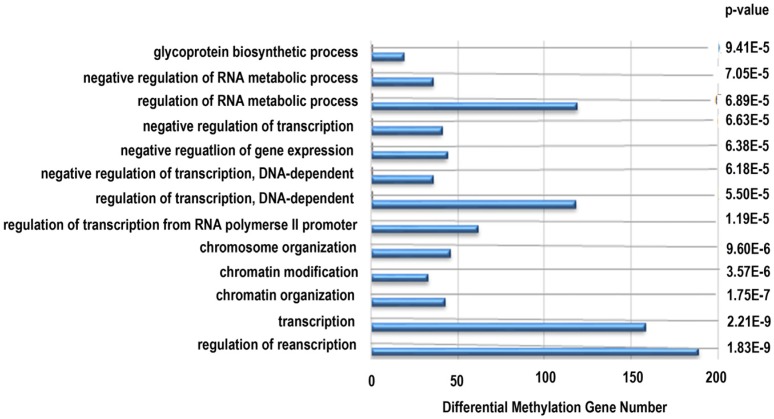
Gene ontology classifications in biology process for hypomethylated genes. The gene ontology (GO) term is on the *y*-axis, number of genes is on the *x*-axis, and the *p*-value indicating significance of enrichment is on the right side.

**Figure 5:**
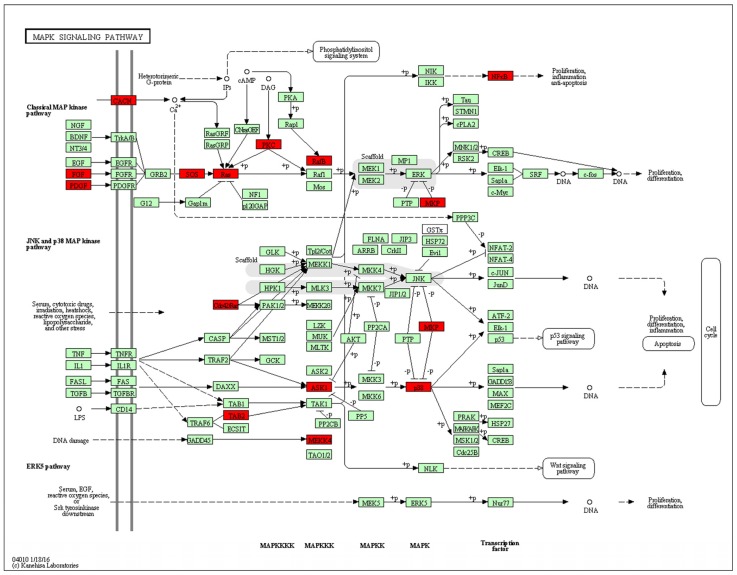
Promoter-specific DNA methylation of genes in the Kyoto Encyclopedia of Genes and Genomes (KEGG) mitogen-activated protein kinase (MAPK) signaling pathway. The red box indicates hypomethylated gene promoters and the map was provided by KEGG Pathway Database.

Next, functional analysis of hypomethylated genes was performed. Function analysis revealed that hypomethylated genes were involved in the regulation of transcript, transcription, chromatin organization, and so on (Table 4, Figure 4). Biological pathway impacted by hypomethylated genes include gap junction and the mitogen-activated protein kinase (MAPK) signaling pathway (Table 5). Figure 5 and Table 6 illustrate that the MAPK signaling pathway in the LC group affected genes exhibiting promoter hypomethylation in the LC group; this was in contrast to what was observed in the CC group.

#### 3.2.7. Bisulfite Sequencing (BSP)

**Figure 6:**
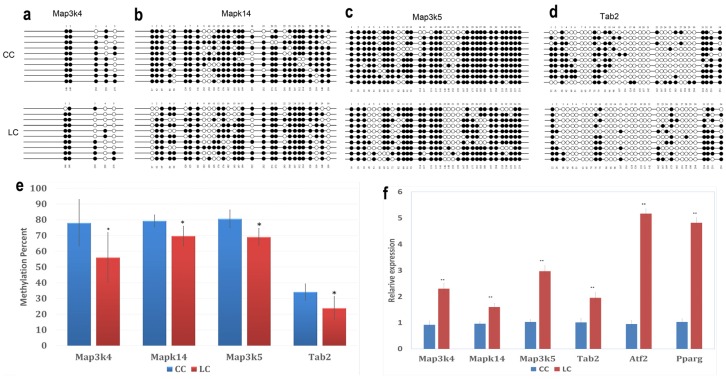
Effects of maternal low chromium diet on methylation of *Map3k4* (**a**); *Mapk14* (**b**); *Map3k5* (**c**); and *Tab2* (**d**) and relative expression (**f**) of genes in adipose of offspring. For bisulfite sequencing (BSP) results, white circles represent unmethylated CpGs, and black circles represent methylated CpGs (**a**–**d**); figure e shows the analysis of DNA methylation status. Values are expressed as means ± SD, *n* = 8 per group. For BSP, pooled DNA of eight mice adipose for each group is what we used for analysis. For each gene in each group, 10 clones randomly selected were analyzed and represent the average methylation level for the indicated genes. * *p* < 0.05, **p < 0.01, vs. CC. *Map3k4*: mitogen-activated protein kinase kinase kinase 4; *Mapk14*: mitogen-activated protein kinase 14; *Map3k5*: mitogen-activated protein kinase kinase kinase 5; *Tab2*: TGF-β-activated kinase 1/MAP3K7 binding protein 2; *Pparg*: peroxisome proliferator activated receptor gammas; *Atf2*: activating transcription factor 2.

Bisulfite Sequencing (BSP) on four hypomethylated promoters was performed. Five to 35 CpGs for each gene were involved in the analysis. The methylation percentages of these promoters are shown in Figure 6a–e. Mitogen-activated protein kinase kinase kinase 4 (*Map3k4*), mitogen-activated protein kinase kinase kinase 5 (*Map3k5*), mitogen-activated protein kinase 14 (*Mapk14*), and TGF-β-activated kinase 1/MAP3K7 binding protein 2 (*Tab2*) were significantly hypomethylated in the LC group, compared to the CC group (*p* < 0.05). This result confirmed the results acquired using the DNA methylation array.

#### 3.2.8. Real-Time RT PCR Assay for BSP-Validated Hypomethylated Genes and Downstream Genes

To explore whether hypomethylated promoters can affect gene expression, the expression of four genes (*Map3k4*, *Map3k5*, *Mapk14*, and *Tab2*) regulated by these hypomethylated promoters and two downstream genes (*Atf2*, *Pparg*) was analyzed by real-time PCR. The results showed that the LC group had significantly high expression levels of *Map3k4*, *Map3k5*, *Mapk14*, *Tab2*, *Atf2*, and *Pparg* compared to CC group (*p* < 0.01, Figure 6f).

## 4. Discussion

Most importantly, in this study we found that body weight was comparable among the different groups at birth and at 3 weeks of age. However, at 32 weeks, pups from low chromium diet dams were heavier than those of the control group. Switching to the control diet did not correct the body weight increase. Other studies have reported that maternal chromium restriction significantly increased body weight from 12 months of age. Rehabilitation regimes did not correct the change [16]. It has also been reported that dietary vitamin B_12_ restriction increased body weight from weaning [23].

In our study, food intake was comparable among all groups at 32 weeks. However, maternal chromium restriction increased fat pad weight in offspring at 32 weeks of age. Reversing the diet did not correct this change. Venu et al. found that offspring from vitamin-restricted dams had a significantly higher percentage of body adipose from day 100 [24].

In addition, maternal chromium restriction may increase serum TG and LDL in offspring at 32 weeks of age; reversing the diet corrected LDL levels only. Other groups have found that maternal vitamin or chromium restriction may also significantly increase circulating triglyceride levels [16,24].

Moreover, maternal chromium restriction may increase serum TNF-α levels. TNF-α modulates lipid metabolism and is associated with obesity. The long-term effect of maternal magnesium restriction increases plasma TNF-α in offspring at 18 months [25]. Several groups found that maternal protein restriction upregulated TNF-α expression in the liver and spleen, and increased serum TNF-α from fetal age to adulthood [26,27,28]. 

Early nutritional exposures can affect later disease risk through the epigenomic changes (DNA methylation, histone modifications, and so on). Although these epigenomic changes do not directly affect the DNA nucleotide sequence, it can be passed on to subsequent generations [8]. DNA methylation, which usually occurs at cytosines within CpG islands, is a key regulator in gene transcription. CpG islands are usually located in or near the promoters of the genes [29,30]. The DNA methylation changes in promoter regions are negatively related to gene expression [31].

**Figure 7:**
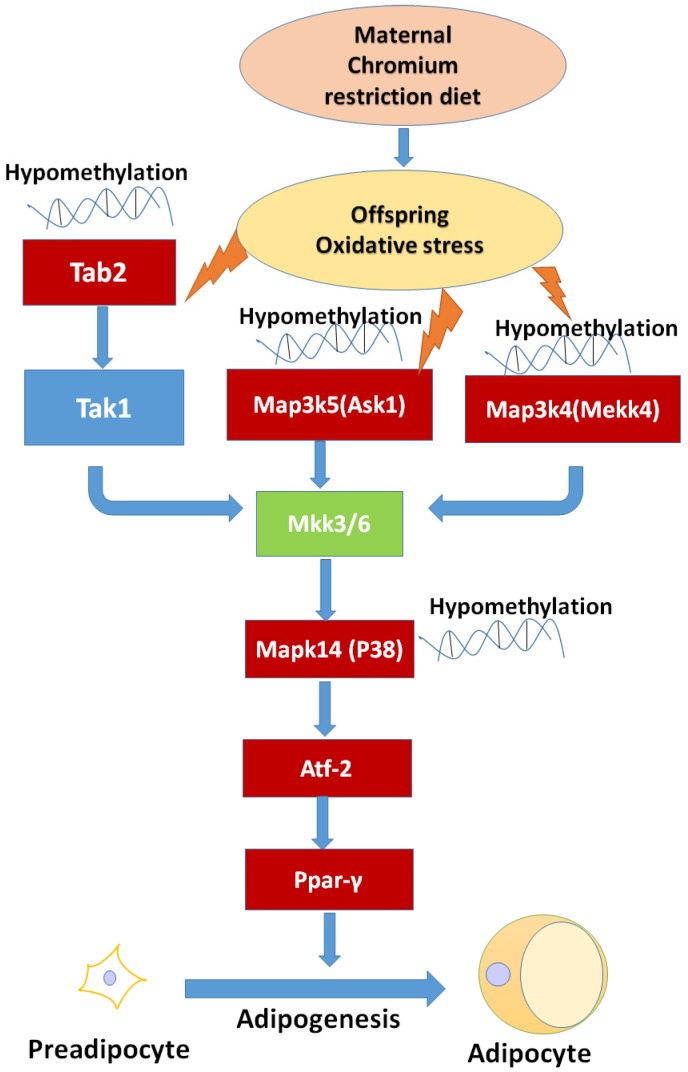
The possible mechanism of maternal chromium restriction on offspring. Maternal chromium restriction induces oxidative stress, then leads to hypomethylate in Map3k5 (Ask1), *Tab2*, Map3k4 (Mekk4), Mapk14 (p38 MAPK) promoter, activates Ppar-γ through Atf-2 to accelerate adipogenesis in adipose of mice offspring. The red box indicates hypomethylated gene promoters, and upregulated gene expression. Map3k4: mitogen-activated protein kinase kinase kinase 4; *Mapk14*: mitogen-activated protein kinase 14; *Map3k5*: mitogen-activated protein kinase kinase kinase 5; *Tab2*: TGF-beta activated kinase 1/MAP3K7 binding protein 2; *Tak1*: Transforming growth factor-β-activated kinase 1; *Ask1*: apoptosis signal-regulated kinase 1; Mkk3/6: mitogen-activated protein kinase kinase 3/6; *Pparγ*: peroxisome proliferator activated receptor gammas; *Atf2*: activating transcription factor 2.

After genome-wide screening for differentially methylated promoters, we found that MAPK signaling was located at the center of many different methylated genes (Figure 7), such as *Map3k4*, *Mapk14*, *Map3k5*, *Tab2*. MAPKs can regulate many cellular processes, such as normal cell proliferation, differentiation, and apoptosis [32]. More and more evidence shows that MAPK-dependent signal transduction is related to adipogenesis [33,34]. The MAPK family consists of three main members: extracellular signal-regulated protein kinases (ERKs), c-Jun N-terminal kinases (JNKs), and p38 kinases. Inflammatory cytokines and many other cellular stresses may activate the p38 MAPK pathway. The p38 MAPK takes part in apoptosis and cell cycle regulation [35]. Transforming growth factor-β-activated kinase 1 (TAK1) belongs to the MAPK family [36]. TAK1 activity is regulated by TAB1, TAB2, TAB3, and TAB4 [37,38]. The activated TAK1 induces the activation of p38 MAPK. Liu et al. found TNF-α markedly promotes the interaction between TAB2 and TAK1, leading to the phosphorylation of p38 MAPK. Pre-treatment with docosahexaenoic acid (DHA) can block the interaction between TAB2 and TAK1 and can attenuate the TNF-α-induced phosphorylation of ERK [39]. We found that maternal low chromium diet could lead to the demethylation of TAB2 and p38, and activation of *Tab2* and p38 expression. Finer et al. found that maternal gestational diabetes leads to genome-wide DNA methylation difference in the placenta and cord blood of exposed offspring. Pathways involved in MAPK signaling are enhanced in methylation genes [40]. *Nyggf4*, which is a newly discovered obesity candidate gene, may regulate the methylation levels of various isoforms of the MAPK subfamily [41].

MAPK family is sensible to imbalance of antioxidant system [42]. Our research found that maternal chromium restriction increased serum MDA level and reduced GSH/GSSG ratio in offspring. Indeed, the measurement of oxidative stress and antioxidant markers is often disturbed by interference from the assay or overestimation derived form stressing analysis conditions. However, MDA is an end-product of lipid hydroperoxide and a widely used marker of the status of oxidative stress [43]. And GSH is a critical antioxidants in vivo. It can neutralize reactive oxygen species through converting to GSSG. Reduction of the GSH/GSSH ratio is an important biomarker of oxidative stress [44]. Saben et al. identified pathways affected by oxidative stress significantly increased in placenta from obese women [45]. Maternal low protein diet increases oxidative stress and reduces anti-oxidant enzyme activity in placenta and fetal livers of rats [46]. Oxidative stress is one mechanism programming offspring metabolic outcomes.

P38 MAPK can activate PPARγ through activating transcription factor-2 (ATF-2) [47,48]. PPARγ is a key transcription factor medicating adipocyte differentiation [49]. Our result also showed that maternal chromium restriction activate the expression of *Atp2* and *Pparg* in adipose of mice offspring. Activated *Pparg* induces the expression of lipolytic (lipoprotein lipase) and lipogenic (fatty acid synthase) enzymes, which modulate fatty acid uptake and synthesis [50,51]. Increased PPARγ levels are observed in the adipose tissue of obese animals and humans [52,53,54]. Ahmad et al. found a significant upregulation of hepatic PPARγ expression in 12 months old rat pups born to vitamin B12-deficient mothers [55].

## 5. Conclusions

In summary, this study is the first to demonstrate a linkage among maternal low chromium diet, the regulation of methylation levels in MAPK pathway genes, and dysfunctional lipid metabolism in offspring. This could lead to adipogenesis and lipogenesis and could contribute to an enhanced susceptibility to the development of obesity in later life. Identifying the biological underpinnings of these mechanisms could help us find early interventional treatments for offspring due to maternal undernutrition. Based on the results revealed by this study, the MAPK pathway genes methylation could be considered as a possible target to modulate the development of lipid dysfunction in the offspring born from mothers with chromium restriction diet during pregnancy.

## Figures and Tables

**Table 1:** PCR Primers for bisulfite sequencing.

Gene	Accession Number	Primer Sequences (from 5′ to 3′)	Production Size	CpG Number
*Map3k4*	NM_011948	F: 5′-AATGATTGAAAGATGTTTTGTT-3′	303	5
		R: 5′-TTCATAAACTAAAACTCAAAATCTC-3′		
*Mapk14*	NM_001168513	F: 5′-GGAGTAAGTAGGGTGGTTTTGT-3′	382	30
		R: 5′-CAAACTTTACCCTACAACTCCTC-3′		
*Map3k5*	NM_008580	F: 5′-GTAAGGGAGTTGTTGYGGAGTA-3′	258	35
		R: 5′-AAAAAAACAAAACTTCCTCCTCTT-3′		
*Tab2*	NM_138667	F: 5′-TGTTGATTAAGGAAAGTTTAGYG-3′	252	34
		R: 5′-CRAAACCCTACAAACCCTAAC-3′		

PCR: Polymerase Chain Reaction; *Map3k4*: mitogen-activated protein kinase kinase kinase 4; *Mapk14*: mitogen-activated protein kinase 14; *Map3k5*: mitogen-activated protein kinase kinase kinase 5; *Tab2*: transforming growth factor-β (TGF-β)-activated kinase 1/MAP3K7 binding protein 2.

**Table 2:** Primer using in real time PCR.

Gene	Accession Number	Primer Sequences (from 5′ to 3′)	Production Size
*Map3k4*	NM_011948	F: 5′-ATTGGAGAAGGACAGTAT-3′	107
		R: 5′-ATAGTCTTGTGGTCGTTA-3′	
*Mapk14*	NM_001168513	F: 5′-TGTTCTGTCTATCTCACTTC-3′	75
		R: 5′-GAGGCACTTGAATGGTAT-3′	
*Map3k5*	NM_008580	F: 5′-AATAATGAAGTTGAGGAGAAGACA-3′	78
		R: 5′-AGAGGAAGCACCGAAGTT-3′	
*Tab2*	NM_138667	F: 5′-TATCAGTGCTTGGAATGG-3′	143
		R: 5′-GACCTTCTTAACGCTCAT-3′	
*Pparg*	NM_001127330	F: 5′-GCATCAGGCTTCCACTAT-3′	75
		R: 5′-CTTCAATCGGATGGTTCTTC-3′	
*Atf2*	NM_001025093	F: 5′-GGCGTTCAAGCAGGATTC-3′	106
		R: 5′-TGACACTGAGACCATAGCAATA-3′	

*Map3k4*: mitogen-activated protein kinase kinase kinase 4; *Mapk14*: mitogen-activated protein kinase 14; *Map3k5*: mitogen-activated protein kinase kinase kinase 5; *Tab2*: TGF-β-activated kinase 1/MAP3K7 binding protein 2; *Pparg*: peroxisome proliferator activated receptor gammas; *Atf2*: activating transcription factor 2.

**Table 3:** Body weight, fasting blood glucose and serum chromium level in dams at weaning.

Groups	C	L
Body weight (g)	23.1 ± 3.5	22.3 ± 3.4
Serum chromium (ng/mL)	0.89 ± 0.22	0.45 ± 0.09 **

C, control diet group; L, low chromium diet group. Values are mean ± SD, *n* = 8 per group. ** *p* < 0.01 vs. C.

**Table 4:** Annotation of LC group hypomethylated promoter genes (*p* value < 0.00001).

Term	Term Number	Count	*p*-Value	Fold Enrichment
Biological process	-	-	-	-
regulation of transcription	GO:0045449	189	$1.83\times 10^{-9}$	1.5015
transcription	GO:0006350	158	$2.21\times 10^{-9}$	1.5775
chromatin organization	GO:0006325	43	$1.75\times 10^{-7}$	2.4151
chromatin modification	GO:0016568	33	$3.57\times 10^{-6}$	2.4737
chromosome organization	GO:0051276	46	$9.60\times 10^{-6}$	2.0145
regulation of transcription from RNA polymerase II promoter	GO:0006357	62	$1.19\times 10^{-5}$	1.7807
regulation of transcription, DNA-dependent	GO:0006355	118	$5.50\times 10^{-5}$	1.4250
negative regulation of transcription, DNA-dependent	GO:0045892	36	$6.18\times 10^{-5}$	2.0679
negative regulation of gene expression	GO:0010629	44	$6.38\times 10^{-5}$	1.8987
negative regulation of transcription	GO:0016481	41	$6.63\times 10^{-5}$	1.9500
regulation of RNA metabolic process	GO:0051252	119	$6.89\times 10^{-5}$	1.4149
negative regulation of RNA metabolic process	GO:0051253	36	$7.05\times 10^{-5}$	2.0546
glycoprotein biosynthetic process	GO:0009101	19	$9.41\times 10^{-5}$	2.8731
Cellular componts	-	-	-	-
nuclear lumen	GO:0031981	86	$2.10\times 10^{-8}$	1.8592
intracellular organelle lumen	GO:0070013	101	$8.79\times 10^{-8}$	1.7017
organelle lumen	GO:0043233	101	$1.00\times 10^{-7}$	1.6972
endomembrane system	GO:0012505	58	$2.07\times 10^{-7}$	2.0695
membrane-enclosed lumen	GO:0031974	102	$2.64\times 10^{-7}$	1.6585
Golgi apparatus	GO:0005794	68	$3.70\times 10^{-7}$	1.9034
nucleoplasm	GO:0005654	57	$1.54\times 10^{-5}$	1.8165
Golgi apparatus part	GO:0044431	29	$2.07\times 10^{-5}$	2.4388
Golgi membrane	GO:0000139	20	$9.16\times 10^{-5}$	2.7868
Molecular Function	-	-	-	-
DNA binding	GO:0003677	167	$1.62\times 10^{-12}$	1.6952
transcription regulator activity	GO:0030528	113	$1.85\times 10^{-8}$	1.6939
transcription factor activity	GO:0003700	76	$1.22\times 10^{-6}$	1.7706
metal ion binding	GO:0046872	260	$8.05\times 10^{-5}$	1.2209
cation binding	GO:0043169	262	$8.06\times 10^{-5}$	1.2192

**Table 5:** KEGG pathway in LC group hypomethylated genes (*p* < 0.01).

Pathway Name	Pathway Term	Gene Count	Fold Enrichment	*p*-Value
Gap junction	mmu04540	11	2.6025	0.008845
MAPK signaling pathway	mmu04010	23	1.7660	0.009097

**Table 6:** LC group hypomethylation genes in MAPK signaling pathway (peak score > 2).

Gene Name	Accession	Chromosome	Peak to TSS	Promoter Classification	Peak Score	Peak M Value
Rac1	NM_009007	chr5	264	HCP	2.42	0.550
Rela	NM_009045	chr19	349	HCP	2.65	0.477
Prkcb	NM_008855	chr7	350	HCP	2.96	0.370
Cacna1h	NM_001163691	chr17	37	HCP	3.1	0.338
Map3k4	NM_011948	chr17	−422	HCP	3.48	0.334
*Tab2*	NM_138667	chr10	−175	HCP	3.68	0.312
Fgf3	NM_008007	chr7	336	HCP	2.08	0.305
Braf	NM_139294	chr6	105	HCP	2.48	0.265
Mapk14	NM_001168513	chr17	−833	HCP	3.38	0.259
Mapk14	NM_001168514	chr17	393	HCP	3.38	0.259
Fgf18	NM_008005	chr11	85	HCP	2.93	0.258
Map3k5	NM_008580	chr10	−291	HCP	2.6	0.254
Cacna2d3	NM_009785	chr14	−314	HCP	2.94	0.251
Pdgfa	NM_008808	chr5	−301	HCP	3.42	0.247
Fgf8	NM_010205	chr19	−231	HCP	2.59	0.218
Cacnb4	NM_001037099	chr2	145	ICP	2.27	0.169
Fgf11	NM_010198	chr11	117	ICP	2.11	0.144
Hras1	NM_001130444	chr7	−254	HCP	2.36	0.121
Sos1	NM_009231	chr17	−350	HCP	2.49	0.109
Cacna1g	NM_001177890	chr11	−401	HCP	2.15	0.060
1500003O03Rik	NM_019769	chr2	437	HCP	2.37	0.053
Dusp16	NM_130447	chr6	−410	HCP	2.18	0.038
Prkx	NM_016979	chrX	−253	HCP	2.22	0.035
Nfkb2	NM_001177370	chr19	−536	ICP	2.1	0.012

TSS, transcription start sites; Peak to TSS, the distance from the center of the peak to the TSS. (“-”: peak center in upstream of the TSS); Peak Score, the average of −log_10_*^p^*^−value^ from the probes within the peak. The score reflects the probability of positive enrichment. (cut-off = 2); Peak M Value, the median of log_2_^−ratio^ from the probes within the peak. The score reflects the methylation degree; HCP, high CpG promoters/regions; ICP, intermediate CpG promoters/regions; LCP, low CpG promoters/regions.
